# Isolation of *Monascus purpureus* HC-5 and Optimization of Solid-State Fermentation for High-Yield Pigment Production

**DOI:** 10.3390/microorganisms13122874

**Published:** 2025-12-18

**Authors:** Wenli Quan, Shuanglian Chen, Han Li, Zhen Tang, Mohammad Nur Alam, Xun Liu

**Affiliations:** 1 School of Food and Liquor Engineering, Sichuan University of Science & Engineering, Yibin 644000, China; quanwenli0426@163.com (W.Q.); 18140267032@163.com (S.C.); 324086002210@stu.suse.edu.cn (H.L.); 323086002218@stu.suse.edu.cn (Z.T.); 2 Bangladesh Wheat and Maize Research Institute, Nashipur, Dinajpur 5200, Bangladesh; nur.alam@bwmri.gov.bd

**Keywords:** *Monascus* pigments, *Monascus purpureus*, response surface methodology, solid-state fermentation

## Abstract

*Monascus* pigments (MPs) are valuable natural colorants, but their industrial production is often hampered by scarce high-yield strains and inefficient processes. In this study, a novel MPs-producing strain was isolated from red yeast rice and named as *Monascus purpureus* HC-5. This strain exhibited notable hydrolase activities, indicating a high efficiency in substrate utilization. In addition, using rice as the optimal substrate, the key parameters of solid-state fermentation were optimized. Response surface analysis revealed that soaking time and temperature were the most critical influencing factors. The optimal combination conditions were an inoculum size of 8.91%, a soaking time of 13.59 h, and a temperature of 32 °C. Under these optimized conditions, the MPs yield reached 2185 ± 255.7 U/g, which displayed an approximately four-fold increase compared to the initial unoptimized process. Briefly, this study identified a strain of *Monascus purpureus* and optimized its solid-state fermentation conditions, which significantly increased the yield of MPs. This provides an important theoretical basis and scientific evidence for the large-scale industrial production of MPs.

## 1. Introduction

As a group of small filamentous fungi, *Monascus* spp. have been widely utilized in various fields, including food fermentation, medicine and health care in China for over a thousand years [1]. The primary metabolites of *Monascus* include various enzymes, such as esterase, glucoamylase and acid protease [2]. These enzymes act in synergy to generate flavor compounds and volatile aromatic substances, which not only boost the quality of alcoholic drinks but also enrich their color and taste, thus promoting the extensive application of *Monascus* in the brewing sector [3]. In addition, *Monascus* species are capable of producing a variety of secondary metabolites, including *Monascus* pigments (MPs), lovastatin, γ-aminobutyric acid (GABA), and ergosterol [4]. MPs are safe and non-toxic natural pigments, and have been currently used in food additives and healthcare products [5,6].

**Figure 1:**
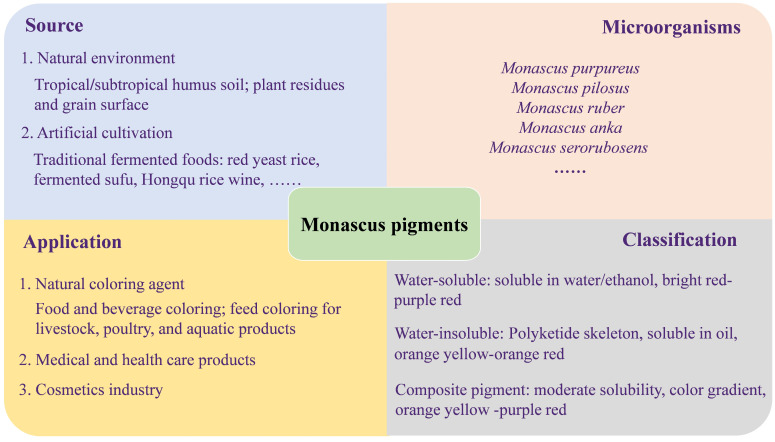
The source, application and classification of MPs.

MPs are produced by *Monascus* through solid-state or liquid fermentation and belong to natural edible pigments [7]. The strains that have been reported capable of producing MPs include *Monascus purpureus*, *M. ruber*, *M. anka*, and *M. pilosus* (Figure 1). *Monascus* strains produce a variety of pigment compounds that can be classified into yellow, orange and red types based on their color characteristics [8,9]. These pigments have aroused people’s research interest due to their diverse chemical structures and properties [10].

During food processing, the color of food products usually undergoes changes; therefore, colorants are commonly used in industrial production to alleviate this phenomenon. However, safety concerns regarding synthetic pigments still exist, particularly regarding the misuse of unapproved colorants or the excessive intake of certain permitted colorants [11,12]. These concerns, coupled with growing consumer demand for clean-label products, have driven the search for natural alternatives. Compared with synthetic colorants, natural pigments are to some extent safer, healthier, and have a greater market demand [13]. Studies have reported that MPs and their derivatives exhibit various in vitro and in vivo physiological activities at effective doses, such as lipid-lowering effects [14,15], anti-inflammatory [16], prevention of diabetes [17] and Alzheimer’s disease [18], suggesting their potential health benefits beyond coloring. These characteristics make MPs an ideal alternative to chemically synthesized pigments in the food, cosmetics, and pharmaceutical industries, and the market demand has been steadily increasing in recent years.

**Figure 2:**
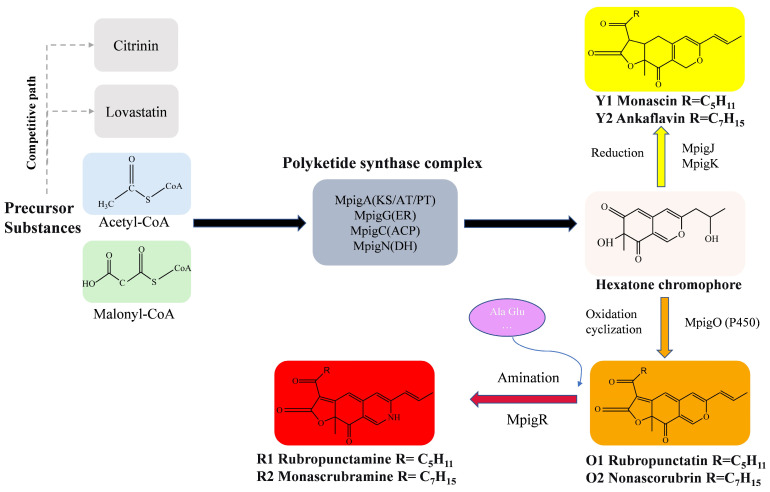
The biosynthesis pathway of MPs.

The biosynthesis of MPs follows the polyketide pathway, using acetyl-CoA and malonyl-CoA as precursors (Figure 2), which has been extensively studied in recent years [8]. Key enzymes and regulatory mechanisms in this pathway have been progressively elucidated, revealing strain-specific differences in gene expression and metabolic regulation that account for variations in pigment yield and composition among different *Monascus* strains [19,20].

The industrial-scale production of MPs still faces difficulties. First, the scarcity of high-yield strains is one of the core factors. The pigment-producing capacity of *Monascus* strains exhibits intraspecific and interspecific variations, and wild-type strains are prone to degeneration after multiple subcultures, leading to decreased production [21]. The value of superior strains lies not only in their final yield but also in their unique physiological and biochemical characteristics. These traits collectively determine the strain’s adaptability to the fermentation environment and its efficiency in substrate conversion [22]. For example, amylase activity helps the efficient utilization of starch in cereal substrates, providing sufficient carbon sources for the rapid growth and vigorous metabolism of strains [20]. Therefore, systematic physiological and biochemical characterization of strains is an important basis for evaluating their industrial application potential and guiding subsequent process optimization.

In addition, the low efficiency of the fermentation process is another critical issue. Solid-state fermentation, which simulates the natural growth environment of *Monascus*, is considered the most suitable method for producing traditional MPs, offering advantages such as low energy consumption, high product concentration, and minimal waste generation [23]. In addition to the challenges in terms of yield and process efficiency, the synthesis of MPs may also be accompanied by the production of citrinin, a nephrotoxic mycotoxin that poses a significant threat to food safety [21,23]. Therefore, alongside high productivity, the selection of microbial strains that inherently produce low or undetectable levels of citrinin is a critical prerequisite for the safe industrial application of MPs.

The selection of fermentation substrate is crucial for solid-state fermentation, as it not only provides nutrients for microbial growth but also serves as a physical support matrix. Rice has a balanced nutritional composition (rich in starch and moderate in protein) and loose structure facilitating mycelial penetration, is regarded as a classical substrate for MPs production [24]. In addition, grains such as wheat and millet have been utilized due to their similar nutritional profiles [25]. Legumes, such as soybeans and mung beans, are rich in protein and can promote the synthesis of red pigments [26]. Furthermore, to enhance economic viability, studies have explored the use of low-cost agricultural by-products or wastes (e.g., bran, soybean residue, bagasse) as alternative fermentation substrates [27,28]. These substrates are generally less expensive than rice and contribute to waste valorization. However, the application effectiveness of these alternative substrates is highly dependent on the inherent characteristics of the strain itself. Different strains, owing to variations in their enzymatic systems, exhibit different capacities for substrate degradation and utilization, consequently resulting in variations in pigment production.

Based on the above research, this study collected traditional fermented food red yeast rice samples, aiming to isolate high-yield MPs-producing strains. Physiological and biochemical experiments were comprehensively employed to systematically analyze the strain’s growth characteristics and key influencing factors of MPs synthesis, providing additional references for current metabolic mechanism research. The key parameters were preliminarily explored through single-factor experiments, and a mathematical model was established by applying the Response Surface Methodology (RSM) to optimize the fermentation conditions. This study will provide excellent strain resources and efficient technical solutions for MPs production.

## 2. Materials and Methods

### 2.1. Experimental Materials and Culture Media

Red yeast rice samples were supplied by an online commercial supplier and were identified as originating from Gutian, Fujian Province, China. Potato dextrose agar (PDA) medium and potato dextrose broth (PDB) medium were used for the isolation and activation of strains, respectively [29]. The preparation of seed culture medium was based on the method reported by Yang et al. [30] with modifications (glucose 30 g/L, peptone 10 g/L, KH_2_PO_4_ 1 g/L, MgSO_4_·7H_2_O 1 g/L, natural pH). These reagents were purchased from Shanghai Aladdin Biochemical Technology Co., Ltd. (Shanghai, China). The spore concentration should be 1 × 10^7^ spores/mL before it can be used for inoculation. Rice (Fragrant rice, Hanzhong, China) solid-state fermentation medium (30 g rice, soaked with water at a 1:1 ratio for 10 h) was used for MPs production. After soaking, the surplus water was drained off, and the moisture content of the prepared substrate was approximately 55% (*w*/*w*). In all fermentation conducted in this study, the same rice variety was adopted to ensure the fermentation medium supplied identical initial nutrients for fermentation.

### 2.2. Isolation and Characterization of MPs-Producing Strains

Under aseptic conditions, red yeast rice was ground into powder and homogenized with 90 mL of sterile 0.9% (*v*/*v*) NaCl solution [31]. The mixture was then cultured at 30 °C, 150 rpm for 5 h. After enrichment, the suspension was diluted and spread onto PDA solid medium, which was subsequently incubated at 30 °C for 48 h. Orange/orange-red single colonies with distinct morphological characteristics were picked and subjected to streak purification. Following three repetitions of the aforementioned steps, vigorously growing single colonies were selected. By repeating these procedures more than three times, single colonies with different morphologies can be selected, numbered, and stored at 4 °C for future use. Strain activation was achieved by inoculating these isolated single colonies into PDB liquid medium and incubating for 48 h at 30 °C with shaking at 150 rpm. Based on characteristics such as the depth of the bacterial suspension color and pigment diffusion range, strains with vivid colors and large diffusion ranges were selected for identification.

The genomic DNA of the selected strain was extracted by using a fungal DNA genome extraction kit from Solarbio (Beijing Solarbio Science & Technology Co., Ltd., Beijing, China). PCR amplification was performed using universal primers, ITS1 and ITS4 [31]. After electrophoresis verification, the PCR products were submitted to Qingke Biotechnology Co., Ltd. (Chengdu, China) for sequencing. The sequencing results were compared with microbial sequences in NCBI, and sequences with high homology were chosen. A phylogenetic tree was constructed in MEGA 12 using the neighbor-joining method with 1000 bootstrap replicates.

### 2.3. Extraction and Determination of MPs

The fermentation products were dried in an oven at 40 °C and pulverized into powder [32]. Pigments were extracted from the total solid fermentation product, including both fungal biomass and rice substrate. According to the experimental method described by Li et al. [1], 1.0 g sample of the ground and sieved powder was weighed into a 100 mL volumetric flask and dissolved with 10 mL of 80% (*v*/*v*) ethanol solution. The mixture was extracted at room temperature for 1 h, then 80% (*v*/*v*) ethanol was added to the original volume, mixed thoroughly, and filtered through filter paper. The filtrate was diluted to maintain the absorbance value between 0.8 and 1.0. The 80% (*v*/*v*) ethanol solution was used as the blank control, and absorbance values were measured at 505 nm (OD_505nm_) to calculate the color value (U/g) using the following formula: Color Value (U/g) = (OD_505nm_ × Dilution Factor × Extraction Volume (mL))/(Dry Sample Weight (g)). The color value was expressed in U/g of dry substrate, where one unit (U) is defined as the absorbance value at 505 nm under the described extraction conditions.

### 2.4. Physiological and Biochemical Characterization

Colony morphology (color, texture, margin) of the target strain on PDA plates, mycelial morphology, and spore characteristics were examined under an optical microscope (Motic China Group Co., Ltd., Xiamen, China). Temperature gradients (15 °C, 25 °C, 30 °C, 37 °C), NaCl gradients (0%, 2%, 5%, 8%, 10% *w*/*w*), and pH gradients (3.0, 5.0, 7.0, 9.0) were established to determine single colony diameter/biomass dry weight of the strain under different conditions. Enzymatic activities were measured by spectrophotometry and expressed in U/mL. The activities of amylase and cellulase were determined by the DNS method, which is used to quantify the amount of reducing sugars released from starch and carboxymethyl cellulose, respectively [33,34]. Protease activity was measured using the Folin-phenol method with casein as the substrate.

### 2.5. Solid-State Fermentation Substrate Selection

Solid-state fermentation was performed in Erlenmeyer flasks (250 mL), with 30 g of pre-prepared rice substrate placed in each flask. After inoculation, the flasks were sealed with breathable membrane seals and incubated statically at the designated temperature. To maintain high humidity and prevent the substrate from drying out, the flasks were placed in an incubator with a relative humidity of >90% (controlled by a built-in humidifier) throughout the fermentation period. Subsequent solid-state fermentation experiments also used the same procedure.

Rice, mung bean, and soybean were selected as initial fermentation substrates to investigate the effects of different fermentation substrates on MPs color value yield. The substrate with the optimal yield was selected as the fermentation substrate for subsequent experiments.

### 2.6. Single-Factor Experimental Design

Single-factor experiments were first conducted to determine the appropriate factor levels and their ranges for the subsequent RSM optimization, ensuring the experimental design was both efficient and focused on the relevant parameter space.

Inoculum size, soaking time, and cultivation temperature were chosen as individual factors to examine their impacts on the color value yield. Using the controlled variable method, inoculum sizes were set at 6%, 8%, 10%, 12%, and 14% (with temperature at 30 °C and soaking time of 10 h); soaking time were set at 5 h, 10 h, 15 h, 20 h, and 25 h (with inoculum size of 8% and temperature at 30 °C); temperature were set at 25 °C, 28 °C, 30 °C, 32 °C, and 37 °C (with constant inoculum size of 8% and soaking time of 10 h). The optimal parameter range was determined based on the color value yield of MPs in the fermentation products. The inoculum size was expressed as (*v*/*w*)%, referring to the volume (mL) of the standardized spore suspension per 100 g of dry substrate.

### 2.7. Box–Behnken Design

Using the Box–Behnken design method, fermentation conditions were optimized through a three-factor, three-level trials. Drawing on the results of single-factor experiments, three factors affecting MPs color value yield (inoculum size, soaking time and temperature) were incorporated into the experimental design. Table 1 shows the codes and levels for each factor. RSM was adopted to assess and optimize the solid-state fermentation conditions, thereby determining the optimal fermentation conditions for MPs production on rice solid medium. A total of 17 experimental runs with 5 center points were conducted for response surface analysis. A regression equation was derived through multiple regression analysis, while three-dimensional response surface plots were constructed using Design-Expert 13.0.

### 2.8. Data Analysis

Three biological replicates and three technical replicates were set for each experiment in the present study. Data recorded during the experimental process were preliminarily organized using Excel 2021. Phylogenetic tree construction was completed using MEGA 12.0 software. Data visualization was carried out in Origin 2024 software.

## 3. Results

### 3.1. Isolation of MPs-Producing Strains

Collectively, five strains were acquired from red yeast rice samples and numbered as HC-1 to HC-5. Through morphological observation and pigment analysis, it was found that one strain (HC-5) could produce a relatively high content of MPs, so the HC-5 strain was selected for subsequent experiments.

**Figure 3:**
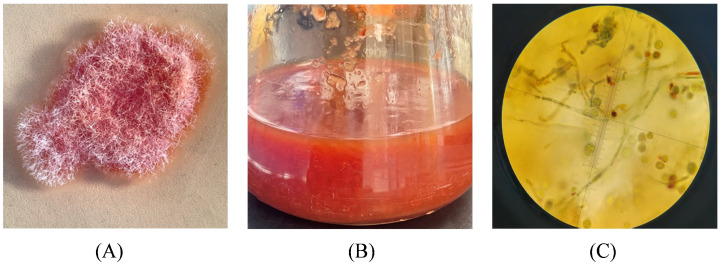
Morphological characteristics and phylogenetic tree construction of strain HC-5. (**A**) Strain HC-5 on PDA solid medium; (**B**) Strain HC-5 in PDA liquid medium; (**C**) Morphology of HC-5 strain under 100× optical microscope, with septate hyphae and oval spores.

As shown in Figure 3A, the colony morphology of HC-5 was orange-red with a dry surface, producing white mycelium with a loose texture and presenting a delicate cobweb-like structure that was difficult to pick, with irregular margins. The morphology of strain HC-5 in the liquid culture medium is shown in Figure 3B. Under optical microscopy, the mycelia of the red mold were observed to be septate, exhibiting an irregular branched structure with conidia that were approximately elliptical in shape (Figure 3C). Based on the above observations, strain HC-5 was preliminarily identified as belonging to the genus *Monascus*.

**Figure 4:**
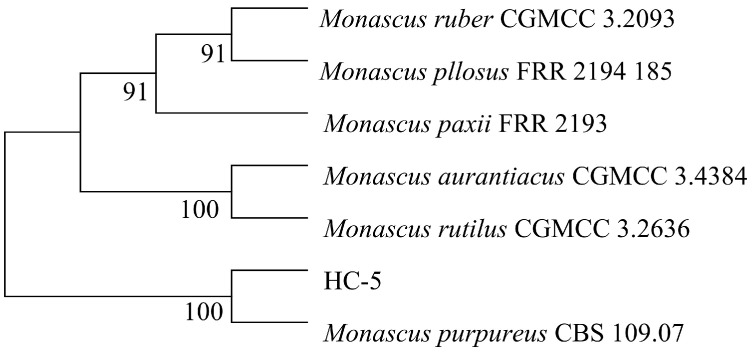
The constructed phylogenetic tree of strain HC-5.

The ITS sequencing results of strain HC-5 were compared with other species, and a phylogenetic tree was built. The finding demonstrated that strain HC-5 shared the closest phylogenetic relationship with *Monascus purpureus* CBS109.07 (Figure 4). Therefore, it was named as *Monascus purpureus* HC-5 (HC-5).

### 3.2. Physiological and Biochemical Characteristics of HC-5 Strain

**Figure 5:**
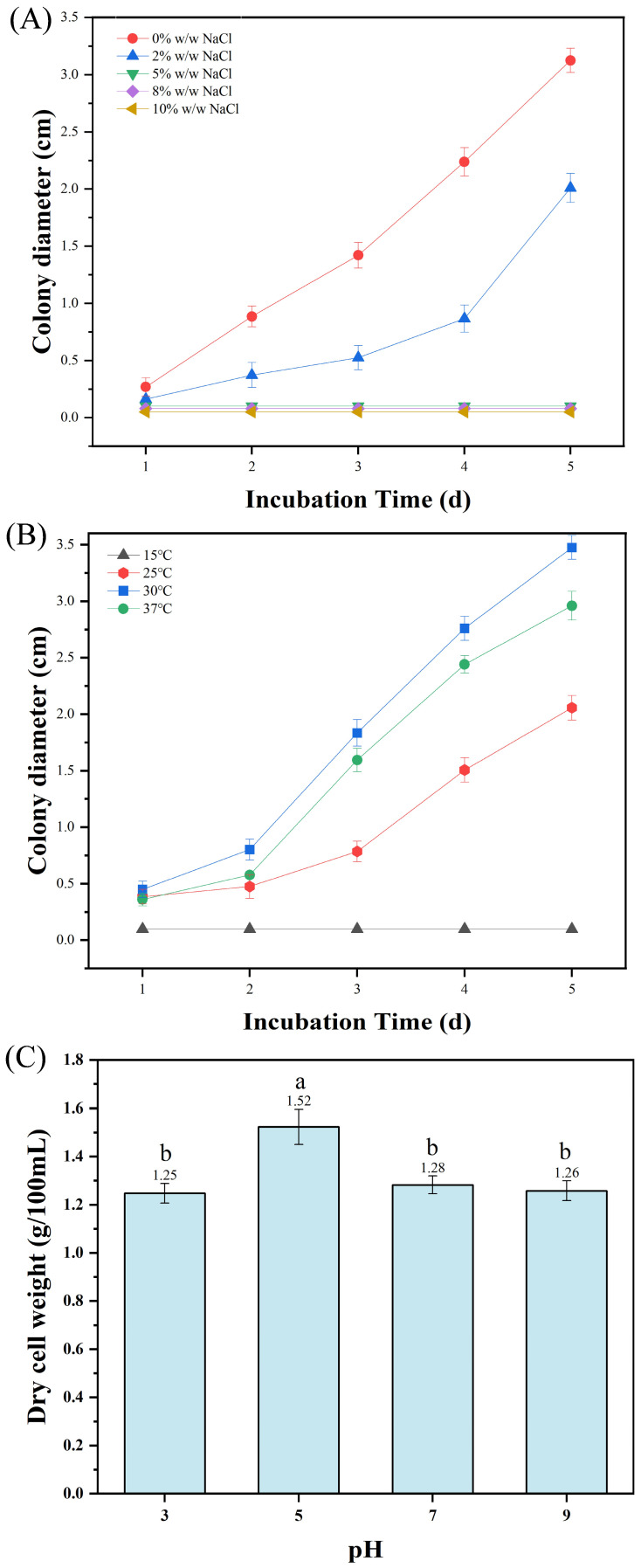
Physiological and biochemical characteristics of strain HC-5. (**A**) Salt tolerance; (**B**) Temperature adaptability; (**C**) pH adaptability. Data are expressed as mean ± SD, and the different letters on the bar chart denote statistically significant differences among groups at *p* < 0.05 (one-way ANOVA).

To investigate the physiological and biochemical characteristics of HC-5 strain, experiments on salt tolerance, temperature and pH adaptability were conducted (Figure 5). The results showed that the colony diameter was largest under the control (0% NaCl), followed by that under 2% NaCl treatment. Other higher salt concentrations visibly decreased the colony diameter of the strain (Figure 5A). For the temperature adaptability, the colony diameter at 30 °C was obviously larger than that at other temperature. Both excessively high and low temperature were not conducive to the growth of colonies (Figure 5B). When the pH was 5, the dry cell weight was significantly higher than that at other pH values (Figure 5C). In addition, the strain HC-5 exhibited amylase activity of 3.817 ± 0.393 U/mL, protease activity of 0.828 ± 0.091 U/mL, and cellulase activity of 0.109 ± 0.009 U/mL, indicating that it has a certain degree of ability to degrade carbohydrate, protein, and cellulose.

### 3.3. Selection of the Optimal Solid-State Fermentation Substrate

**Figure 6:**
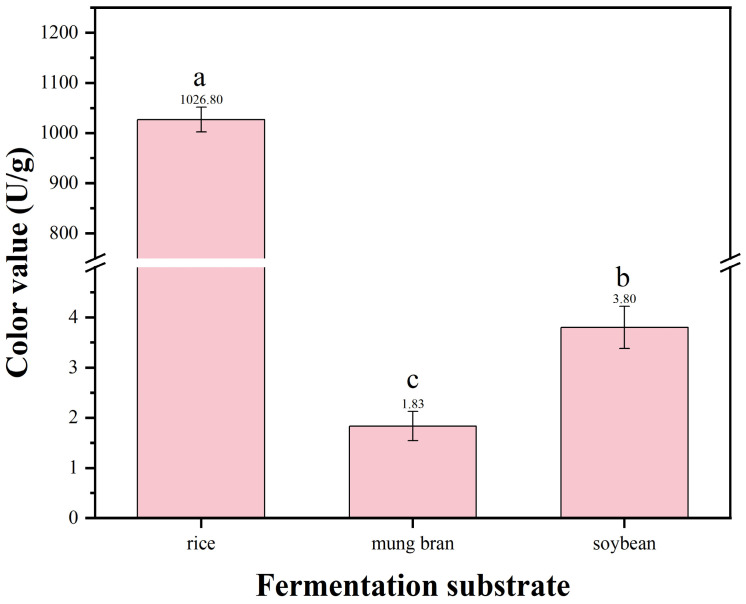
Color value of MPs in different fermentation substrates. Data are expressed as mean ± SD, and the different letters on the bar chart denote statistically significant differences among groups at *p* < 0.05 (one-way ANOVA).

Rice represents a carbon source-dominant substrate, rich in starch, capable of providing sufficient and readily available nitrogen sources and energy for the growth of *Monascus*, serving as a traditional carbon-type substrate and positive control. Soybean is a high-nitrogen substrate that can provide abundant nitrogen sources and lipid precursors for *Monascus* cell growth and metabolism. Mung bean is a carbon-nitrogen balanced substrate that can simultaneously provide good carbon and nitrogen sources for *Monascus*, potentially facilitating the coordination between cell growth and secondary metabolite synthesis. Therefore, rice, mung beans and soybeans were selected as substrates to determine the optimal solid-state fermentation medium for the HC-5 strain (Figure 6).

The results showed that using rice as the substrate led to the highest color value of MPs produced by strain HC-5 (Figure 6). In the rice medium, the yield of pigment reached 1026.8 ± 109.86 U/g, which was significantly higher than that in mung bean and soybean medium (Figure 6). Therefore, rice was selected as the fermentation substrate for subsequent experiments.

### 3.4. Single-Factor Analysis

#### 3.4.1. Effect of Inoculum Size on MPs Production

**Figure 7:**
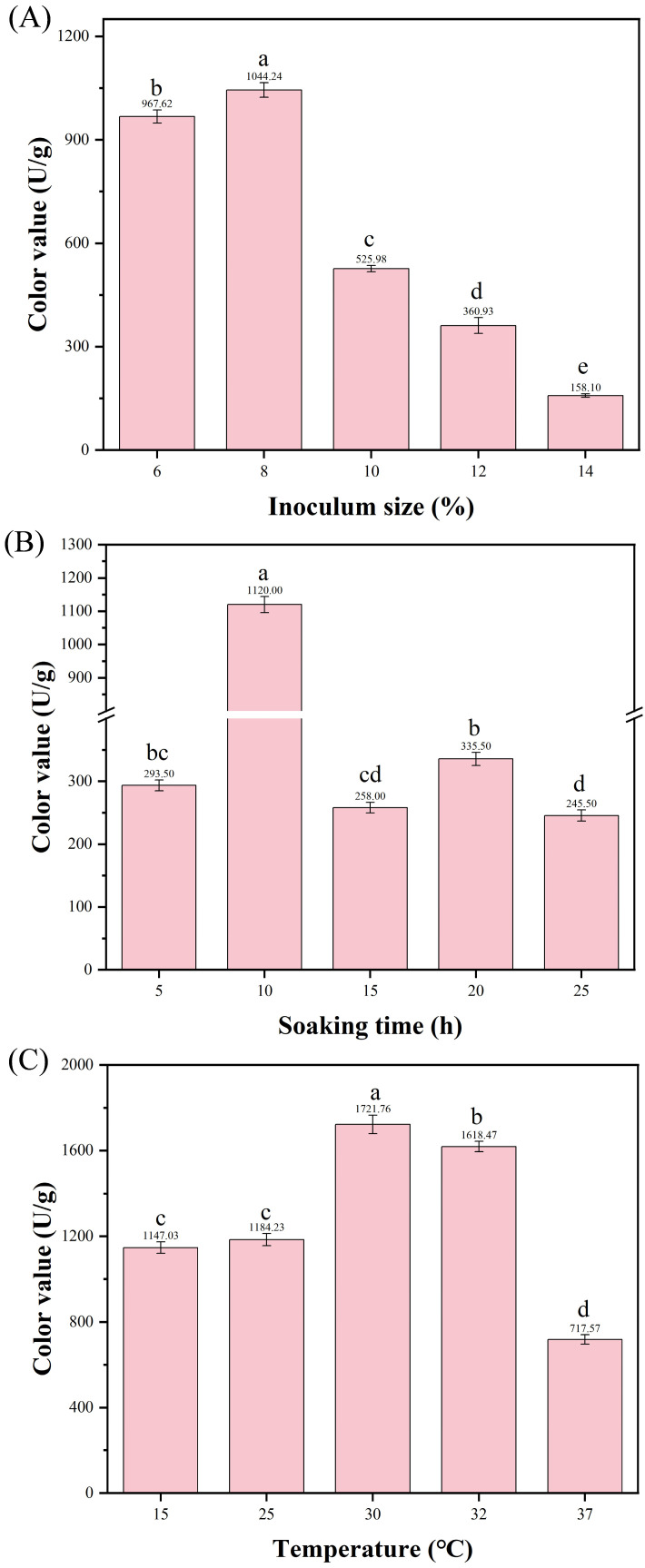
Effects of single factors on the color value of MPs. (**A**) Inoculum size (with fixed temperature of 30 °C and soaking time of 10 h); (**B**) Soaking time (with fixed inoculum size of 8% and temperature of 30 °C); (**C**) Temperature (with fixed inoculum size of 8% and soaking time of 10 h). Data are expressed as mean ± SD, and the different letters on the bar chart denote statistically significant differences among groups at *p* < 0.05 (one-way ANOVA).

Inoculum size is a critical factor affecting fermentation efficiency and yield. An appropriate inoculum size ensures that the strain rapidly establishes substrate dominance, shortens the fermentation cycle, and effectively inhibits microbial contamination [35]. To ensure balanced cell growth and vigorous metabolism, selecting an appropriate inoculum size is crucial for MPs production. At an inoculum size of 8%, the color value of MPs was the highest (1044.24 ± 107.53 U/g) (Figure 7A). Therefore, the inoculum size of 8% was considered optimal.

#### 3.4.2. Effect of Soaking Time on MPs Production

Appropriate moisture content in solid substrates is beneficial for microbial growth and metabolite production. Insufficient moisture content affects microbial absorption and utilization of nutrients in solid substrates, while excessive moisture content causes particle agglomeration, leading to a reduction in dissolved oxygen [36]. Soaking time is a key operational variable for controlling the moisture content of the rice substrate [36]. Therefore, the effect of different soaking times on the fermentation of strain HC-5 was investigated. As illustrated in Figure 7B, the yield of MPs exhibited a trend of initial increase followed by a decrease with the extension of soaking time. When the soaking time was 10 h, the yield of MPs (1120 ± 123.2 U/g) was extremely significantly higher than that under other soaking times. Therefore, 10 h was determined as the optimal soaking time for rice as a fermentation substrate to produce MPs.

#### 3.4.3. Effect of Fermentation Temperature on MPs Production

Diverse microorganisms exhibit distinct temperature requirements for their various metabolic processes. Temperature affects the growth and metabolism of microorganisms in the fermentation system. Inappropriate temperatures can result in uneven fermentation, poor oxygen transfer, and low pigment yield [37]. Therefore, the effect of different fermentation temperatures on MPs production by strain HC-5 was investigated. As the fermentation temperature increased, the yield of MPs first rose and then decreased. At a fermentation temperature of 30 °C, the color value of MPs was the highest, reaching 1731.73 ± 177.33 U/g (Figure 7C). Therefore, 30 °C was considered as the optimal fermentation temperature.

### 3.5. Response Surface Analysis

Drawing on the results of single-factor trials, the Box–Behnken design was adopted to optimize the fermentation conditions for MPs biosynthesis, with inoculum size, soaking time, and temperature served as controlled factors. The color value of MPs was used as the response value, and the experimental data are illustrated in Table 2. A total of 17 tests were included in the response surface design: 12 factorial experiments and 5 center point experiments.

The multivariate regression fitting analysis was performed using the data in Table 2. The response surface regression formula was acquired as follows: Y = 2814.57 + 45.69A + 113.75B + 128.61C + 16.06AB + 42.41AC + 64.69BC − 1104.58A^2^ − 664.58B^2^ − 305.18C^2^.

According to the *p*-values, the effects of single factors on MPs production indicated that B (soaking time) and C (temperature) had a highly significant effects on MPs yield, while A (inoculum size) had a significant effect on MPs yield, but it was less than that of B and C (Table 3). The constructed regression model had a *p*-value < 0.001, demonstrating the regression model was extremely significant. The lack-of-fit *p*-value (0.6904) exceeded 0.05, demonstrating that the pure error was nonsignificant. In addition, the principal factors (A, B, and C) and interaction factors (AC and BC) had significant effects on the yield of MPs (*p* < 0.05), while the quadratic factors (A^2^, B^2^, and C^2^) were also had extremely significant effects on the yield of MPs (*p* < 0.01). These suggested that the relevant factors exerted a substantial implication for MPs production in the rice medium. The coefficient of determination, R^2^ = 0.9989, indicated that the regression model had excellent agreement with the reality. The adjusted model of determination (R^2^_Adj_ = 0.9976) demonstrated that all factors included in the regression model had significant effects on MPs production. With R^2^_Adj_ − R^2^_Pred_ < 0.2, the regression model could thoroughly clarify this mechanism, demonstrating its effectiveness and applicability in predicting the impact of various factors on MPs yield. The adequacy of the model was further confirmed by diagnostic plots (Figure S1), which confirmed the normality of residuals (Figure S1A) and a satisfactory correlation between the predicted and actual values (Figure S1B).

### 3.6. Response Surface Interaction

**Figure 8:**
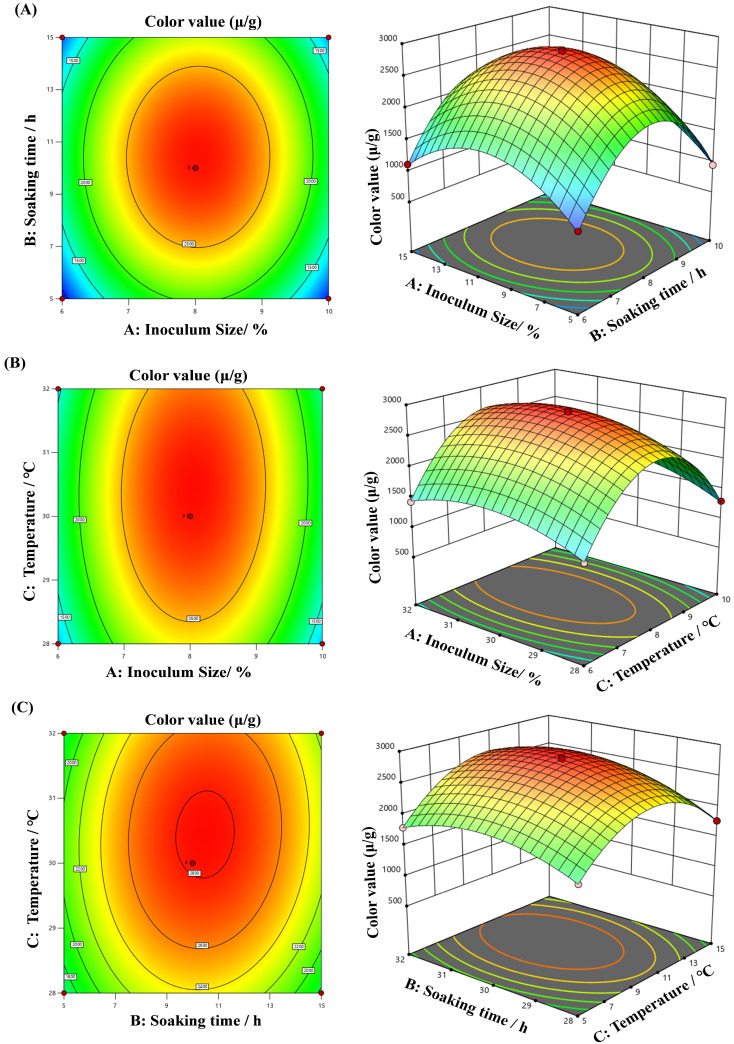
Response surface methodology and contour diagrams for MPs yield. (**A**) Influence of inoculum size and soaking time on MPs yield; (**B**) Influence of inoculum size and temperature on MPs yield; (**C**) Influence of soaking time and temperature on MPs yield.

Integrating response surface analysis and regression equations, three-dimensional surface plots were created using Design-Expert 13.0 software to examine the effects of factor interactions on MPs color value (Figure 8). The results showed that the interaction between inoculum size and soaking time had an effect on MPs color value, but the effect was not significant (Figure 8A). The interactive effect between inoculum size versus temperature had a significant effect on MP color value (Figure 8B). Moreover, the interactive effect among soaking time and temperature had an extremely significant effect on MPs color value (Figure 8C). The statistical relevance of the pairwise interactions presented in Figure 8 aligns with the analysis outcomes of the *p*-values of the interaction items in Table 3. The optimal combination fermentation parameters for MPs yields simulated in this research were as listed below: inoculum size of 8.91%, soaking time of 13.59 h, and fermentation temperature of 32 °C. Under these fermentation conditions, the calculated maximum MPs color value was 2240.43 U/g.

### 3.7. Validation Experiments

The aim of the validation experiment was to confirm the reliability of the regression model under actual conditions. Taking into account the feasibility of practical operations, the fermentation conditions were specified as follows: inoculum size of 9%, soaking time of 13 h, and temperature of 32 °C. Under such circumstances, three replicate experiments were conducted, and the detected MPs yield was 2185.596 ± 255.70 U/g. The discrepancy between this value and the theoretical value was 54.83 U/g, demonstrating that the constructed regression model was valid and could be applied to MPs fermentation production.

## 4. Discussion

Red yeast rice, as a traditional substrate for MPs production, harbors natural microbial communities with superior production potential [38]. The physiological and biochemical characterization of strain HC-5 isolated therefrom revealed high adaptability (optimal growth at 30 °C, pH 5.0) (Figure 5), which is favorable for industrial fermentation. Notably, HC-5 exhibited substantial hydrolytic enzyme activities, which likely played a direct mechanistic role in its superior MPs yield [21]. Specifically, the elevated amylase activity ensures efficient starch degradation, providing a robust supply of acetyl-CoA and malonyl-CoA—the essential precursors for the polyketide synthase (PKS) pathway that synthesizes MPs [8]. Concurrently, the pronounced protease activity liberates amino acids from substrates, which serve as critical amino donors for the transamination reaction that converts orange azaphilone intermediates into stable red pigments [20]. Therefore, the synergistic action of these enzymes in HC-5 potentially enhances carbon flux into the PKS pathway and facilitates the downstream conversion to red pigments, providing a biological basis for the observed optimization results. Additionally, *Monascus* strains with high amylase activity can more effectively utilize inexpensive starchy materials and exhibit a positive correlation with pigment yield, a phenomenon also reported in other studies [39].

The type of fermentation substrate is the primary factor affecting the yield of secondary metabolites. In this study, when rice was used as the substrate, the MPs color value (1026.8 ± 107.8 U/g) was significantly higher than that of mung bean and soybean substrates (Figure 6). The advantage of rice may originate from its unique nutritional composition and physical structure. First, the main component of rice is starch, which provides an efficient and readily available carbon source for *Monascus* growth and metabolism. Second, the loose and porous structure of rice grains facilitates mycelial invasion and growth, providing a larger solid–liquid interface area, thereby promoting the transfer of oxygen and nutrients, which is crucial for aerobic *Monascus* fermentation [40]. In contrast, soybeans and mung beans with higher protein and fat content may not only have lower carbon source supply efficiency, but the ammonia or other intermediate products generated from their catabolism may also exert certain inhibitory or interfering effects on pigment synthesis [22].

The fermentation process was successfully optimized using RSM based on the preliminary conditions determined by single-factor experiments (inoculum size 8%, soaking time 10 h, temperature 30 °C). The established model was highly significant (*p* < 0.0001), demonstrating its reliability in reflecting the intrinsic relationship between factors and pigment yield [41]. Under the optimal conditions (inoculum size 8.91%, soaking time 13.59 h, temperature 32 °C), the validation result (2185.6 ± 255.7 U/g) was in close agreement with the theoretical prediction (2240.43 U/g). This not only validated the accuracy of the model but, more importantly, highlighted the significant advantage of RSM in revealing multi-factor interactions and achieving synergistic optimization. It is particularly noteworthy that the pigment yield achieved by strain HC-5 under this optimized process is significantly higher than that reported in recent comparable studies. For instance, the maximum color value reported on jackfruit seed substrate was approximately 1125 U/g [26], and a value of 1800 U/g was achieved with an optimized corn cob substrate supplemented with soybean oil [42]. Furthermore, the results of this study demonstrate strong competitiveness when compared with the contemporaneous RSM-based optimization by Huang et al. [2]. The high yield of MPs achieved under solid-state fermentation highlights the great potential of strain HC-5 for industrial applications and indicates that the optimization strategy developed in this study is refined and effective, providing a valuable reference for the industrial-scale fermentation of high-yield MPs.

Fermentation temperature (C) was identified as an extremely significant influencing factor (*p* < 0.0001) in this study, primarily because it directly regulated the activity and metabolic rate of various enzymes in the cell (including key enzymes such as PKS in the MPs synthesis pathway). Deviation from the optimal temperature range can lead to a sharp decline in enzyme activity and metabolic disorder [43]. The significant effect of soaking time (B) indicated that achieving an appropriate initial moisture content was crucial for rice substrates, as it allowed rice grains to fully absorb water and soften, facilitating starch gelatinization and mycelial penetration and growth, thereby improving substrate accessibility and bioconversion efficiency. Insufficient moisture leads to mass transfer difficulties, while excessive moisture may cause nutrient loss and increase the risk of contamination. The strategy of optimizing moisture content through pretreatment to promote microbial fermentation has been widely proven effective in solid-state fermentation research [44]. In this study, the interaction term BC (soaking time × temperature) exhibited extreme significance (*p* = 0.0081) (Figure 8), which indicated that the effect of temperature on MPs synthesis changes with soaking time, and vice versa. With a shorter soaking time, insufficiently softened rice grains may require a more suitable temperature to maintain higher enzyme activity to overcome mass transfer resistance; whereas with a longer soaking time, the substrate becomes softer, physical barriers to cell growth and metabolism are reduced, and the effect of temperature may change accordingly.

This study employed response surface methodology to precisely optimize fermentation conditions, significantly improving MPs yield. However, this study also has certain limitations. Firstly, regarding strain identification, although ITS sequencing provided reliable species identification for *M. purpureus* HC-5, future studies could employ multi-locus phylogenetic analysis for more in-depth taxonomic resolution. Secondly, the safety of the produced MPs is crucial for industrial applications. This study primarily focused on the optimization of MPs production processes and did not monitor the dynamic changes in toxic secondary metabolites (such as citrinin). Therefore, subsequent research will not only focus on detecting citrinin under optimized conditions but will also explore strategies to reduce citrinin production. Methods such as genetic modification, process control, or the use of specific inhibitors can be employed to minimize citrinin, ensuring the development of safe and compliant bioproducts. Thirdly, for actual industrial production, this study did not conduct scale-up experiments. Future work should focus on pilot-scale validation and pigment stability. Scaling up solid-state fermentation requires addressing critical challenges such as heat and mass transfer in larger bioreactors [28]. Furthermore, the stability of the extracted pigments under various industrial conditions (e.g., pH, temperature, light) must be thoroughly evaluated to ensure applicability in food and cosmetic products, as their stability can be a limiting factor [45]. Simultaneously, fermentation equipment and processes can be optimized, and online monitoring technology can be introduced to keep track of various parameters and product changes in real time during the fermentation process. Finally, in terms of molecular mechanism research, the expression patterns of key enzyme genes (such as the PKS gene family) in the MPs biosynthesis process were not investigated, making it difficult to elucidate the regulatory mechanisms of MPs synthesis at the gene transcription level. Future research could consider integrating transcriptomics and metabolomics technologies to deeply reveal the molecular regulatory network of MPs biosynthesis from both gene expression and metabolite change perspectives.

## 5. Conclusions

In this study, a high-yield MPs-producing strain was identified and named as *Monascus purpureus* HC-5. In order to find the appropriate conditions for synthesizing MPs, rice was determined as the optimal solid-state fermentation substrate and the effects of inoculum size, soaking time, and temperature on MPs yield were evaluated through single-factor assays. By integrating Box–Behnken design and response surface analysis, a credible regression model was developed, and the optimal solid-state fermentation parameters (after adjusting according to reality) were inoculum size of 8%, soaking time of 10 h, and temperature of 32 °C. Under these fermentation conditions, the MPs yield attained 2185.596 ± 255.70 U/g, approximately four-fold higher than the yield achieved prior to optimization. This research offers a reference for the solid-state fermentation of MPs production. The strain HC-5 and the optimized MPs production process show significant potential for industrial applications, but this potential still needs to be confirmed through further validation, including strict assessment of citrinin levels, scale-up testing, and techno-economic evaluation.

## Figures and Tables

**Table 1:** The codes and levels for each factor in Box–Behnken design.

Factor	Level		
	−1	0	1
(A) Inoculum size (%)	6	8	10
(B) Soaking time (h)	5	10	15
(C) Temperature (°C)	25	30	32

**Table 2:** Box–Behnken experimental design and MPs yield in rice medium.

Test Number	Independent Variables			Color Value (U/g)
	Inoculum Size (%, A)	Soaking Time (h, B)	Temperature (°C, C)	
1	6	5	30	921.68
2	10	5	30	946.19
3	6	15	30	1112.5
4	10	15	30	1201.26
5	6	10	28	1255.24
6	10	10	28	1296.56
7	6	10	32	1428.24
8	10	10	32	1639.19
9	8	5	28	1665.17
10	8	15	28	1767.83
11	8	5	32	1792.41
12	8	15	32	2153.83
13	8	10	30	2832.23
14	8	10	30	2820.68
15	8	10	30	2847.52
16	8	10	30	2826.49
17	8	10	30	2745.93

**Table 3:** Response surface regression model evaluation of deviance.

Source	Sum of Squares	Degrees of Freedom	Mean Square	F-Value	*p*-Value	Significance
Model	8.310 × 10^6^	9	9.233 × 10^5^	738.98	<0.0001	**
A-Inoculum size	16,701.98	1	16,701.98	13.37	0.0081	**
B-Soaking time	1.035 × 10^5^	1	1.035 × 10^5^	82.84	<0.0001	**
C-Temperature	1.323 × 10^5^	1	1.323 × 10^5^	105.91	<0.0001	**
AB	1032.18	1	1032.18	0.8261	0.3936	
AC	7193.58	1	7193.58	5.76	0.0475	*
BC	16,739.18	1	16,739.18	13.40	0.0081	**
A^2^	5.137 × 10^6^	1	5.137 × 10^6^	4111.79	<0.0001	**
B^2^	1.860 × 10^6^	1	1.860 × 10^6^	1488.43	<0.0001	**
C^2^	3.921 × 10^5^	1	3.921 × 10^5^	313.87	<0.0001	**
Residual	8745.81	7	1249.40			
Lack of fit	2457.37	3	819.12	0.5210	0.6904	
Pure error	6288.45	4	1572.11			
Cor total	8.318 × 10^6^	16				
	R^2^ = 0.9989	R^2^_Adj_ = 0.9976	R^2^_Pred_ = 0.9941			

“**” denotes an highly significant difference among the results (*p* < 0.01), and “*” denotes a significant difference among the results (*p* < 0.05).

## Data Availability

The original contributions presented in this study are included in the article/Supplementary Material. Further inquiries can be directed to the corresponding author.

## Supplementary Materials

The following supporting information can be downloaded at: https://www.mdpi.com/article/10.3390/microorganisms13122874/s1, Figure S1: Model diagnostic plots for the response surface regression model.
