# Supplementary material for: Isolation of Monascus purpureus HC-5 and Optimization of Solid-State Fermentation for High-Yield Pigment Production
Source: Microorganisms. 2025 Dec 18;13(12):2874. doi: 10.3390/microorganisms13122874 (PMC12736185; doi:10.3390/microorganisms13122874)
Supplement: Supplementary file 1 [file microorganisms-13-02874-s001.zip › microorganisms-3976106-supplementary.pptx]

## Slide 1
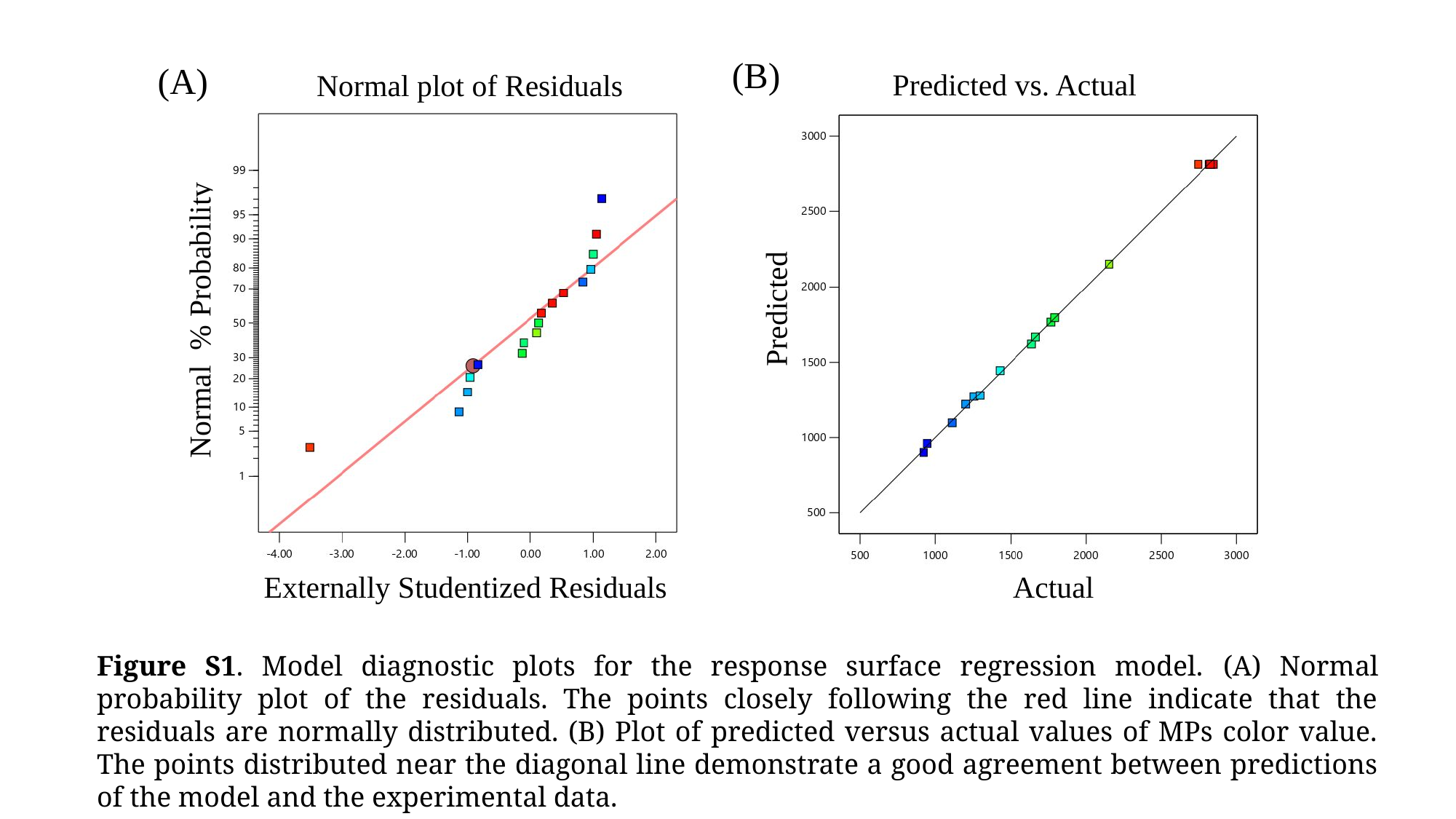

(B)
(A)
Predicted vs. Actual
Normal plot of Residuals
Predicted
Normal % Probability
Actual
Externally Studentized Residuals
Figure S1. Model diagnostic plots for the response surface regression model. (A) Normal probability plot of the residuals. The points closely following the red line indicate that the residuals are normally distributed. (B) Plot of predicted versus actual values of MPs color value. The points distributed near the diagonal line demonstrate a good agreement between predictions of the model and the experimental data.
